# Role of Primary Cilia in Skeletal Disorders

**DOI:** 10.1155/2022/6063423

**Published:** 2022-06-18

**Authors:** Xinhua Li, Song Guo, Yang Su, Jiawei Lu, Donghua Hang, Shao Cao, Qiang Fu, Ziqing Li

**Affiliations:** ^1^Department of Orthopedics, Shanghai General Hospital, Shanghai Jiao Tong University School of Medicine, Shanghai 200080, China; ^2^Department of Joint Surgery, Shandong Provincial Hospital, Shandong University, Jinan 250012, China; ^3^Department of Spinal Surgery, Shanghai East Hospital, Shanghai Tongji University School of Medicine, Shanghai 200120, China; ^4^Department of Joint Surgery, Shandong Provincial Hospital Affiliated to Shandong First Medical University, Jinan 250021, China; ^5^Orthopaedic Research Laboratory, Medical Science and Technology Innovation Center, Shandong First Medical University & Shandong Academy of Medical Sciences, Jinan 250117, China

## Abstract

Primary cilia are highly conserved microtubule-based organelles that project from the cell surface into the extracellular environment and play important roles in mechanosensation, mechanotransduction, polarity maintenance, and cell behaviors during organ development and pathological changes. Intraflagellar transport (IFT) proteins are essential for cilium formation and function. The skeletal system consists of bones and connective tissue, including cartilage, tendons, and ligaments, providing support, stability, and movement to the body. Great progress has been achieved in primary cilia and skeletal disorders in recent decades. Increasing evidence suggests that cells with cilium defects in the skeletal system can cause numerous human diseases. Moreover, specific deletion of ciliary proteins in skeletal tissues with different Cre mice resulted in diverse malformations, suggesting that primary cilia are involved in the development of skeletal diseases. In addition, the intact of primary cilium is essential to osteogenic/chondrogenic induction of mesenchymal stem cells, regarded as a promising target for clinical intervention for skeletal disorders. In this review, we summarized the role of primary cilia and ciliary proteins in the pathogenesis of skeletal diseases, including osteoporosis, bone/cartilage tumor, osteoarthritis, intervertebral disc degeneration, spine scoliosis, and other cilium-related skeletal diseases, and highlighted their promising treatment methods, including using mesenchymal stem cells. Our review tries to present evidence for primary cilium as a promising target for clinical intervention for skeletal diseases.

## 1. Introduction

Primary cilia are highly conserved microtubule-based organelles that project from the cells' surface into the extracellular environment and play important roles in mechanosensation, mechanotransduction, and polarity maintenance during organ development and pathological changes, including in the skeletal system [1, 2]. The assembly and function of cilia require effective intraflagellar transport (IFT) in the cilium, which is a bidirectional transport operated by IFT protein complexes and IFT motors. IFT protein complexes are divided into complex A and complex B [3]. In primary cilia, membrane cargos are trafficked in vesicles to the ciliary base by the Bardet-Biedl syndrome (BBSome) coat complex [4]. The IFT gene or BBS gene mutation can cause cilium defects or loss. Primary cilia have a complex function including transducing Hedgehog signaling and sense and transduce chemical or mechanical signal. The role of cilia in Hedgehog signaling transduction was found by Huangfu et al. in 2003, and the process has been well established [5]. In the absence of ligand, Patched (Ptch) prevents translocation of Smoothened (Smo) to the plasma membrane. A microtubule-associated complex promotes the processing of Gli into its repressor form. Upon activation of the pathway, Smo moves to the plasma membrane, and the Sufu-Gli complex associates with the carboxy-terminal tail of SMO, resulting in the release of Gli and promotion of the processing of Gli into its activated form [6].

The skeletal system consists of bones, cartilage, intervertebral disc (IVD), tendons, and ligaments, providing support, stability, and movement to the body. Skeletal systems are exposed to various mechanical loads and function as a major system for the mechanical transduction in our body. Primary cilia were regarded as a chemical or mechanosensor and signaling pathway transduction center. Therefore, it is believed that cilia have a critical role in skeletal function. In the last several years, emerging studies reported that cells with cilium defects in skeletal systems can cause many human diseases, including osteoarthritis (OA) and intervertebral disc degeneration (IVDD), tendinopathy, Jeune's syndrome, and spinal scoliosis [7–11]. In addition, the primary cilium is well known to play an important role in osteogenic/chondrogenic induction of mesenchymal stem cells (MSCs), regarded as a promising target for clinical intervention for skeletal disorders. New approaches to treat osteoporosis, OA, and other skeletal disorders have focused on promoting bone or cartilage formation through the targeting of osteoblasts/chondrocyte and their progenitors or MSCs. Here, we reviewed available literatures on primary cilia and their role in skeletal disorders and their promising treatment methods, including MSCs.

## 2. Primary Cilia and Ciliary Proteins in Skeletal Diseases

### 2.1. Primary Cilia and Osteoporosis and Fracture Healing

Osteoporosis is one of the most prevalent chronic skeletal pathologic diseases characterized by decreased bone mass, placing an enormous economic burden on patients and payors all over the world [12]. As an exquisitely mechanosensitive organ, mechanical stimulation deficiency has been regarded as a leading cause of osteoporosis. Primary cilia are sensory organelles that play an important role in translating extracellular chemical and mechanical cues into cellular responses and are believed to be closely related to bone development and osteoporosis. The essential role of primary cilia on bone development and patterning has been well established [1, 13, 14] (Table 1). Also, knockout of many cilium-related genes leading to the cilium defects was reported to cause mouse long bone or vertebral osteoporosis phenotype, including IFT80, IFT88, Kinesin family member 3A (Kif3a), Evc, Pkd1, and IFT40 [11, 15–21].

New methods to treat osteoporosis focused on promoting osteogenic induction of MSCs, and the primary cilia were reported to be essential for MSCs' osteogenic differentiation [22]. Corrigan et al. [23] found that LiCl and fenoldopam can be utilized to enhance ciliogenesis in MSCs and fenoldopam is a viable ciliotherapeutic option to enhance MSCs' osteogenesis and potential to treat osteoporosis. However, how the cilium changed in osteoblasts or osteocytes during osteoporosis is largely unknown. Further study to identify the relationship between primary cilia and osteoporosis needed to be investigated.

Fracture healing is a complex biological process that shares some similarity feature with the bone development. Recently, Liu et al. [24] found that conditional deletion of IFT80 in chondrocytes utilizing tamoxifen-inducible Col2-CreER mice resulted in low-density/porous woven bony tissue compared to control during fracture healing. Mechanistically, IFT80 deletion can downregulate the TGF-*β* signaling pathway by inhibiting the expression of TGF-*β*I and TGF-*β*R and phosphorylation of Smad2/3 in the fracture callus. Chinipardaz et al. [25] reported that loss of cilia caused by diabetes in osteoblasts resulted in defective diabetic fracture healing by using in a streptozotocin-induced diabetes and Osx-cre;IFT80^fl/fl^ mouse model. All these demonstrated that cilia are important in bone fracture healing.

### 2.2. Primary Cilia and Bone or Cartilage Tumors

**Figure 1:**
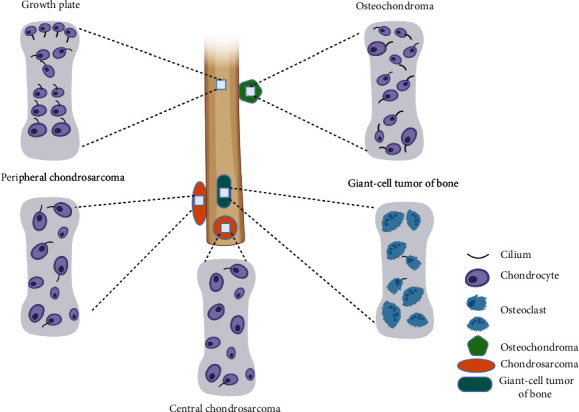
Schematic representation shows the cilium feature in bone or cartilage tumors. In both the proliferative and hypertrophic zones of the normal growth plate, the cilium is well orientated as shown in each layer. However, in osteochondroma, chondrocyte arrangement and cilia orientation are dramatically disorganized. In human malignant chondrosarcomas, cilium incidence is reduced and cilium orientation is disorganized. In the mouse peripheral chondrosarcoma, primary cilium is dramatically reduced and cilium orientation is disorganized. Giant cell tumor of bone is composed of mononuclear stromal cells and numerous macrophage giant cells, but only mononuclear stromal cells of giant cell tumor of bone present primary cilia.

Bone or cartilage tumors are one of the most common human primary bone lesions, and they range from benign lesions, such as enchondromas and osteochondromas, to malignant chondrosarcoma [26]. Enchondromas and osteochondromas are the most common benign bone tumor, and they are always developing during periods of bone growth in a location adjacent to the growth plate [26, 27]. Enchondromas occur within the metaphyseal portion of bone. Osteochondromas manifest as outgrowths of bone and cartilage from the metaphyseal region of long bones, with a cartilage cap on. The development of bone or cartilage tumors is always combined with constitutively active hedgehog (Hh) signaling. The primary cilium is the center for the Hh signaling transduction; thus, the relationship between cilia and osteochondromas and enchondromas has been investigated. In osteochondroma, the primary cilium incidence was normal, but the cilium orientation was dramatically disrupted compared with control [28]. Cilium organization is essential for cells' polarity, and the disorganized cilium orientation in most cells of osteochondromas may contribute to the loss of cell polarity and arrangement in the growth plate [29]. However, the cilium incidence in enchondroma is reported to vary in different studies [28]. Ho et al. [30] reported that only 13.4% of cells are ciliated in enchondroma tissues, which significantly decreased compared with control articular cartilage (Figure 1). Recently, we found that the cilium incidence and cilium length were comparable between human enchondroma cells and control articular chondrocytes, but the cilium orientation largely alters [31]. The different sample resources may contribute to variation in different samples, and the cilium features in more human enchondroma samples are needed to be identified in the future. Chondrosarcoma is a cartilaginous origin malignant tumor with aggressive behavior. In human and mouse chondrosarcomas, the cilium incidence of neoplastic chondrocytes is dramatically lost compared with normal articular cartilage [30]. Parts of chondrosarcoma are thought to originate from benign tumors when combined with P53 mutation [26]. The dramatic decreased cilium incidence from osteochondromas to chondrosarcoma transition suggested that the percentage of ciliated cells can serve as a useful marker to distinguish benign and malignant tumors.

Hedgehog (Hh) concentration distribution gradient is essential for normal chondrocyte proliferation and differentiation. However, the Hh gradient is disrupted and showed a homogeneous pattern in enchondroma or osteochondroma [31]. Activated Hh signaling in the growth plate (Col2*α*1-Gli2 overexpressed mouse) leads to enchondroma in mice. Similar to the Col2*α*1-Gli2 mouse, IFT88 partial mutant also developed enchondroma around the growth plate. Interestingly, activated Hh signaling (Gli2-overexpressed) in the IFT88 deficiency mouse can cause much more enchondromas. The disruption of cilia in Gli2-overexpressed mouse results in much more enchondromas, suggesting that cilia can inhibit Hh signaling activation under these conditions [30]. Some studies [32, 33] suggested that Hh signaling is essential for bone tumor growth and process; loss of primary cilium-disrupted Hh signaling can inhibit tumor growth or process. However, we found that Indian hedgehog (Ihh) ablation in aggrecan-positive progenitors produced enchondroma-like tissues near the growth plates in mice, and smoothen agonists can significantly reduce the enchondroma incidence in Ihh-knockout mouse [31]. Consistently, the dual and opposing roles of primary cilia and Hh signaling were also found in medulloblastoma development [34]. How cilia and Hh signaling are involved in bone tumor development and progress needs to be investigated furtherly.

Ciliogenesis and elongation processes require the coordination of microtubule assembly and protein modification. Histone deacetylase 6 (HDAC6), as a special member of the HDAC family, plays a vital role in microtubule deacetylation [35]. Xiang et al. [35] reported that a significant decrease in cilium expression and abnormal expression of HDAC6 existed in human chondrosarcoma tissues, and targeting inhibition of HDAC6 could significantly suppress chondrosarcoma cell proliferation and invasion. The potential mechanism may affect ciliogenesis via the Aurora A-HDAC6 cascade. Although these in vitro data on the therapeutic effect of HDAC inhibition on chondrosarcoma are promising, the data from the clinical trial are discouraging in patients [36]. Whether HDAC inhibition or other drugs targeting cilia or cilium-related signaling are effective in the treatment of chondrosarcoma remains to be demonstrated in the future [37].

Giant cell tumor of bone, which usually appears in long bone epiphysis in young adults, is a locally aggressive primary bone neoplasm composed of proliferative mononuclear stromal cells, numerous reactive macrophages, and large osteoclast-like multinucleated giant cells. Castiella et al. [38] found that mononuclear stromal cells of giant cell tumor of bone present primary cilia, and the Hh signaling pathway is activated in these cells. They speculated that primary cilia may play an important role in giant cell tumor of bone tumorigenesis and could be used as a potential therapeutic target in the future.

### 2.3. Primary Cilia and OA

**Figure 2:**
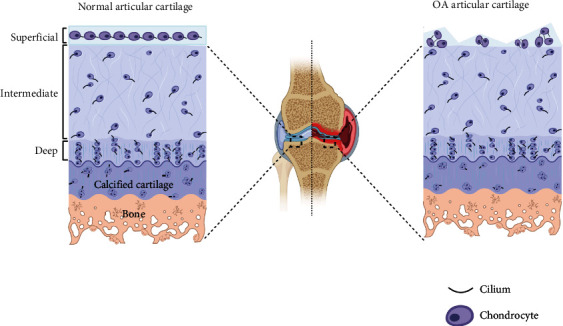
Cilium in normal and osteoarthritis (OA) articular cartilage tissues. The normal articular cartilage can be divided into superficial, intermediate, and deep zones as shown in figures. In the superficial zone of normal articular cartilage (left), the chondrocytes are ellipsoid. Both chondrocyte and cilia are parallel to the surface of articular cartilage. In intermediate and deep zones, the chondrocytes are irregular, but the cilium orientation is on the medial or lateral cell membranes along the longitudinal axis parallel to the chondrocyte. However, the articular surface is eroding in human OA tissue, and the cilium incidence and length significantly increased compared with normal human articular cartilage [40]. Moreover, the cilia are oriented parallel to the long axis of cells at the articulating surface in normal articular cartilage, but it is oriented to the center of abnormal cell clusters in osteoarthritic cells.

OA is one of the most prevalent joint diseases of advanced age and is a leading cause of disability worldwide. OA patients usually suffer from many annoying complications that negatively influence their quality of life. In pathophysiology, OA is characterized by the degeneration of articular cartilage and elevated chondrocyte mortality [39]. Abnormal mechanical overload has been found to be one of the major contributions to the onset and progression of OA. Primary cilia, which have been found crucial in biomechanical signaling transduction, are linked to OA by many studies in the last several years. Primary cilia were found present on both normal articular cartilage and OA tissue, and the cilium incidence and length significantly increased in the eroding articulating surface of human OA compared with normal human articular cartilage [40] (Figure 2). Moreover, the cilia are oriented parallel to the long axis of cells at the articulating surface in normal articular cartilage, but they are oriented to the center of abnormal cell clusters in osteoarthritic cells [40]. Alkaptonuria (AKU) is an inherited disease resulting from a deficiency of the enzyme homogentisate 1,2-dioxygenase which is characterized by severe cartilage degeneration, similar to that observed in OA. However, Thorpe et al. [41] found that the cilium length is dramatically decreased in AKU articular chondrocytes when compared to healthy controls. All these suggested that primary cilia are closely related to OA, but how cilia changed and functioned during this process is still unclear.

To know how cilia functioned during articular cartilage and OA development, different cilium-related genes were deleted by genetic editing technology in the mouse model (Table 2). Deletion of IFT88 in cartilage causes several OA phenotypes with increased expression levels of degeneration markers, including MMP13, collagen type X, Adamts5, and Runx2 [8]. Similarly, the Bardet-Biedl syndrome 1 (Bbs1), Bbs2, or Bbs6 gene mutation mouse model developed OA-like cartilage abnormalities including proteoglycan loss, small surface fibrillation, marked atrophy of the cartilage, and increased MMP13 expression [42, 43]. Moreover, IFT88 deletion following surgical destabilization of the medial meniscus was found to have increased OARSI scores of cartilage damage mouse [43]. All these studies suggested that primary cilia are essential for cartilage development and prevent its degeneration.

Interleukin-1 (IL-1) is one of the most important inflammation media during OA formation and process. Wann and Knight [44] found that IL-1 can elongate the chondrocyte cilia via a PKA-dependent mechanism. Moreover, cilium loss can significantly attenuate IL-1-induced inflammatory response and alleviate the progression of OA. Interestingly, cilium elongation in response to IL-1 requires the accumulation of hypoxia-inducible factor-2*α* (HIF-2*α*) in cilia. Consistently, Yang et al. [45] reported that upregulated HIF-2*α* contributes to OA development through mediating the primary cilium loss. Mechanical stimulation was reported to be anti-inflammatory in many tissues. Recently, Fu et al. [46] reported that mechanical loading can suppress chondrocyte inflammatory induced by IL-1*β* via HDAC6-dependent modulation of tubulin leading to cilium disassembly during OA. Most recently, Fu et al. [47] revealed that osmotic-sensitive ion channel transient receptor potential vanilloid 4 (TRPV4), the key protein for mechanotransduction, localizes to the cilium plasma membrane. Mechanical, osmotic, or pharmaceutical activation of TRPV4 functioned as an anti-inflammatory agent during OA via regulating HDAC6-dependent modulation of ciliary tubulin. These results provided evidence that primary cilium is involved in an inflammatory process and it could be an important target for the treatment of inflammatory diseases such as OA.

Galectin 3 (Gal3) was found to be localized at the cilium base, and its absence causing cilium abnormalities is associated with disrupted epithelial cell polarity. Recently, Hafsia et al. reported that deletion of Gal3 in mouse can develop early onset of OA and exacerbate joint instability-induced OA via mitochondrial apoptosis [48].

### 2.4. Primary Cilia and IVDD

The IVDD occurs in more than 90% of the population older than 50 years [49]. The currently available treatments only provide symptomatic relief from pain [50–53], and these measures cannot decelerate or prevent the progression of degeneration of the intervertebral disc (IVD). Understanding the exact etiology of IVDD and finding the solution to the etiology is the key to cure this disease. A variety of risk factors, such as abnormal mechanical loading, aging, and smoking have been regarded as important factors causing IVDD [54]. Among these factors, abnormal mechanical loading has been considered the major contributor. Although the exact mechanism that abnormal mechanical loads affect cell behaviors in IVD remains unknown, previous studies have revealed that primary cilia played critical roles during cell mechanosensation and mechanotransduction. In the last several years, some scholars have tried to investigate the cilia in IVD and find the existence of primary cilia in IVD. The IVD consisted of nucleus pulposus (NP), annulus fibrosus (AF), and endplate cartilage (EP). Donnelly et al. [55] first attempted to detect cilia in rat IVDs by using multiphoton microscopy and found positive staining in the AF. Zheng et al. [56] examined the primary cilia in the mouse and human NP cells in vitro after 48 h of serum starvation. They furtherly found that parathyroid hormone 1 receptor (PTH1R) is expressed in primary cilia of mouse and human NP cells and knockout PTH1R or cilia in the NP cells result in significant IVDD and blunt the effect of parathyroid hormone on attenuation of aged discs.

**Figure 3:**
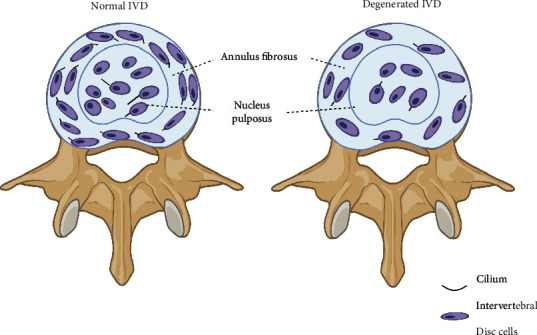
Cilium orientation of the normal and degenerated intervertebral disc (IVD). The IVD consisted of nucleus pulposus (NP), annulus fibrosus (AF), and endplate cartilage (EP). The primary cilia in AF are well organized and orientated: primary cilia were always projected from the inner sides of AF cells (near the NP), and they are oriented parallel to the long axis of the cells. The cilia in NP were disorganized and with varied cilium length. However, in the degenerated IVD, the cilia are disorganized in AF and cilium length and cilium incidence are reduced in both NP and AF.

Recently, we carefully reported that primary cilia are present in the mouse IVD with the cilia-GFP and ARL13B-mCherry;Centrin2-GFP cilium dual reporter-expressing transgenic mouse model [57, 58]. With these two mouse models, we found that the primary cilium length was 0.5-15 *μ*m in the NP and 0.5-3.5 *μ*m in the AF. There are 33.62% of NP cells and 36.1% of AF cells that were ciliated in the mouse's third and fourth lumbar IVD (Figure 3). The NP is derived from the embryonic node and notochord during the development process. Leftward-directed fluid flow, which is produced in embryonic node cilium movement, was regarded as essential for left-right axis determination in mice. Consistently, in our previous study [58], about 2% of cilia with the irregular movement were identified in mouse NP. However, the cilium movement type is different from the clockwise movement of cilia in embryonic notochord/node cells [59]. Interestingly, with the ARL13B-mCherry;Centrin2-GFP mouse model, we find that the cilia in AF are well organized and orientated: primary cilia were always projected from the inner sides of AF cells (near the NP), and they are oriented parallel to the long axis of the cells [58]. To further study the function of primary cilia in IVD, we crossed IFT80^fl/fl^ mice with Col2a1-creERT mice and Col1a2-creERT to impair the primary cilia in IVD. As a result, we find that the deletion of IFT80 can cause an early onset of the IVDD phenotype, characterized by disorganized and decreased growth plate, EP, internal AF (IAF), less compact and markedly decreased gel-like matrix in the NP, and disorganized outer AF (OAF) with thinner, loosened, and disconnected fiber alignment. All these demonstrated that the primary cilia are essential for the maintenance of IVD development [57].

It was reported that NP in IVD can adapt to their physiologically hyperosmotic microenvironment and mediated osmoregulation through the nuclear factor of activated T cell 5 (NFAT5), a tonicity-responsive enhancer-binding protein. As an osmosensor in the natural world, whether cilia contribute to NP cell osmoadaptive response in IVD remains unknown. Choi et al. [60] found that primary cilia in NP cells could change their length in response to osmotic stimulation. However, when silencing of IFT88 or Kif3a to impair primary ciliogenesis did not affect hyperosmotic upregulation of TonEBP, then they concluded that primary cilia in NP have not participated for TonEBP-dependent osmoadaptive response.

In addition, we found that primary cilia in NP reduced during aging and injured induced IVDD and significantly increased during repair, indicating that primary cilia are essential for IVD repair or regeneration [57]. Thus, promoting ciliogenesis in AF and NP progenitors could be a promising target in the treatment of IVDD.

### 2.5. Primary Cilia and Idiopathic Scoliosis (IS)

Scoliosis manifests as spine abnormal three-dimensional curvature, and around 10% of all scoliosis are idiopathic [61]. IS are always born with a normal spine, and the abnormal curvature may begin evident in the adolescent during growth, and IS is diagnosed by excluding congenital defects and other causes of abnormal spine curvatures, such as intervertebral disc or vertebral development defects or other syndromes.

The exact etiology of IS is largely unknown due to its phenotypic and genetic heterogeneity. It is believed that heredity, melatonin, and biomechanical factors of the musculoskeletal system play an important role in its occurrence and progress. Among these factors, the research on genetic correlation has been done by many scholars. Although many genome-wide association studies (GWAS) have found some potential locus mutations, no clear and definite biological mechanism for IS has emerged so far. Nowadays, more and more scholars believed that IS is a complex consequence of genetic variations coupled with biomechanical factors that are affected by individual behavioral patterns. As an organ that bears the main force of the body, the contribution of biomechanics to IS is also valued by researchers. Mechanical loading can alter primary cilium incidence, length, and orientation of chondrocytes, and cilium direction is proven to affect the growth direction in growth plates [57]. The disorganized growth plates were also reported as one of the basic pathology changes in IS [62, 63]. Moreover, several human ciliopathies manifested as skeletal disorders, such as asphyxiating thoracic dystrophy syndrome [64]. Interestingly, the fact that asphyxiating thoracic dystrophy syndrome patients combined with scoliosis makes people believe that IS is a ciliopathy and that the genetic architecture of IS may involve cilium function [64].

Grimes et al. [10] revealed that protein tyrosine kinase-7 (ptk7) mutant zebrafish, a faithful genetic model of IS, exhibits ependymal cell cilium development and cerebrospinal fluid flow defects. Transgenic reintroduction of Ptk7 in motile ciliated lineages prevents scoliosis in ptk7 mutants. Oliazadeh et al. [65] found that primary cilia are significantly elongated in bone cells of IS patients. These IS bone cells can differentially express osteogenic factors and mechanosensitive and signaling genes in response to mechanical stimulation, compared with control. Moreover, many scoliosis association genes [66, 67], for example, TBX6 [68, 69], LBX1, GPR126 [70], PAX1 [71–73], POC5 [74], KIF6 [75], PTK7 [76, 77], FGF3 [78–80], SHP2 [81, 82], IFT88 [64, 83], IFT20 [83], Arl13b [83], and Yap [83], are found to be associated with cilium function so far (Table 3). Therefore, primary cilia are though important for IS development. The monocilia, presenting on the ventral surface of the mouse node, play an important role in determining human left-right symmetry. In addition, the high prevalence of right thoracic IS indicated the possible relation between IS and primary cilia. Burwell et al. [67] think that should the leftward nodal flow of morphogens—which affect precursors of the heart, great vessels, and viscera to create “handed asymmetry”—be extended by anomalous genetic/environmental factors to left-sided mesodermal precursors of vertebrae and ribs, an asymmetric skeletal anomaly may be imprinted. Such an anomaly may lead to relative left costovertebral physeal overgrowth that triggers right thoracic IS and anomalous upper limb length asymmetry. Coincidentally, Burwell et al. found that 50% of patients with dextrocardia had curves convex to the right as it showed in primary ciliary dyskinesia [67]. Schlösser et al. [84] found that the prevalence of scoliosis (Cobb > 10 degrees) and significant spinal asymmetry (Cobb 5–10 degree) were 8 and 23%, respectively, in 198 primary ciliary dyskinesia patients. It was further found that the convexity of the thoracic curve is predominantly to the right in normal organ anatomy and to the left in patients with situs inversus totalis after the analysis of the scoliosis of 16 primary ciliary dyskinesia patients. We observed that around 10% of mice developed scoliosis in our cilium gene-knockout mouse model, even all the knockout mice combined with an extremely narrowed cage (unpublished). Although it was also reported that the rib cage abnormal development can result in progressive thoracic scoliosis [85], only around 10% of mice with the same genotyping developed scoliosis suggesting that the environmental factors or other factors may contribute to scoliosis. It will be interesting to investigate the role of cilium biology and environmental factors in the progress of idiopathic scoliosis in the future.

### 2.6. Primary Cilia and Tendon Disease

Tendons play vital roles in transferring our force from muscle to bone. Tendinopathy is a type of tendon injury and chronic tendon disease, and it is highly prevalent but has few treatment methods so far. Tendon bears dynamic tensile mechanical loading in normal conditions. As a mechanical sensitive organelle, the primary cilia have been found to exist in tendon. Primary cilia were observed in 64% of tenocytes in 3-week-old Sprague-Dawley rats, and they were aligned parallel to the collagen fibers and the long axis of the tendon [86].

Fang et al. [87] found that cilium incidence of tendon enthesis cells increased significantly between postnatal from 4.6% in one week old to 29.7% in two weeks old, and it decreased to 12.1% at 13 weeks old. However, they found a low level of ciliogenesis during the mouse postnatal stage in tendon midsubstance cells. To further know the role of primary cilia in tendon development, the IFT88^fl/fl^ mouse was crossed with Scx-cre mouse for deletion of IFT88 in Scx-expressing cells. As a result, the growth of IFT88-knockout mice was slower, and it showed significantly lower body weights compared to controls. The tendon entheses had decreased structural properties (maximum force and stiffness) and increased material properties (stress and modulus) with drastically smaller cross-sectional areas in tendon entheses in 13-week-old IFT88-knockout mouse, which is an important feature for tendinopathy [88]. Considering that physical loading is an important driver of tendon formation or enthesis pathologies, primary cilia can be promising targets whose mechanosensitivity could potentially be tuned to prevent the progression of tendinopathy. However, how the cilia changed in the tendon during tendinopathy is still largely unknown. Further study on cilia of tendinopathy should be investigated in the future.

### 2.7. Primary Cilia and Other Skeletal Disorders

Ciliary gene mutation can impair skeletal development and cause a group of rare inherited chondrodysplasias diseases. All ciliary chondrodysplasias are characterized by developmental skeletal defects, mainly affecting limbs, ribs, spine, and craniofacial skeleton. They can be subdivided into different groups of severity, clinical phenotype, and underlying genetic defects.

#### 2.7.1. Short-Rib-Polydactyly Syndromes

It is a group of perinatal lethal skeletal dysplasia characterized by severe narrowing of the thorax leading to pulmonary hypoplasia, short limbs, and polydactyly. It is caused by NEK1, DYNC2H1, and other gene mutations [89].

#### 2.7.2. Oral-Facial-Digital Syndrome

Oral-facial-digital syndrome is characterized by pre- and postaxial polydactyly of the hands and feet, tibia hypoplasia, and oral and facial defects. Mutations in TCTN3 may cause up to 50% of all cases [90].

#### 2.7.3. Asphyxiating Thoracic Dystrophy

Asphyxiating thoracic dystrophy (Jeune's syndrome) is characterized by a variable degree of rib shortening, typical pelvis configuration with trident acetabular roof, and acetabular spurs and rarely exhibits polydactyly. Asphyxiating thoracic dystrophy usually is caused by mutations in DYNC2H1, IFT40 and IFT80 [7, 91].

#### 2.7.4. Mainzer-Saldino Syndrome

Mainzer-Saldino syndrome is characterized by cone-shaped epiphyses of the hand, retinal disease, and deterioration of renal function. A narrow ribcage, craniosynostosis, and liver involvement can present in some cases. Causative mutations in IFT140 have been identified in this disease [92].

#### 2.7.5. Cranial-Ectodermal Dysplasia

Cranial-ectodermal dysplasia is a combination of dolichocephaly due to craniosynostosis of the sutura sagittalis, epicanthus, very thin, sparse, and slow-growing hair, tooth abnormalities, brachydactyly, and short rib. Cranial-ectodermal dysplasia is genetically heterogeneous with causative mutations found in IFT122, IFT43, WDR19, and WDR35 [93].

#### 2.7.6. Ellis-van Creveld Syndrome

Ellis-van Creveld syndrome (EVC) is characterized by acromelic dwarfism, polydactyly of the hands' dysplastic nails, tooth abnormalities, and cardiac defects. Biallelic causative mutations in EVC1 and EVC2 have been identified with mutations in EVC1 accounting for 75% and mutations in EVC2 accounting for 25% of the cases [94].

#### 2.7.7. Weyers Acrofacial Dysostosis

Weyers acrofacial dysostosis (Curry-Hall syndrome) is characterized by a milder phenotype of polydactyly, dentition anomalies, and dystrophic nails. Dominant mutations in EVC1 and EVC2 have been found to cause Weyers acrofacial dysostosis [95].

## 3. Conclusion

Numerous studies have shown a variety of functional and structural relationships between primary cilia and physiological as well as pathological aspects of the skeletal system. In this review, we provide insight into the role of primary cilia in skeletal disease and show evidence that the primary cilia may be a promising target of clinical intervention for bone/cartilage tumor, OA, IVDD, scoliosis, osteoporosis, and cilium-related skeletal disease.

## Figures and Tables

**Table 1:** The role of primary cilia in bone development illustrated by the conditional knock out mouse model.

Gene	Function
IFT20	Col1-CreERT;IFT20^fl/fl^ and Osx-Cre;IFT20^fl/fl^ mice exhibit reduced bone mass and strength. Deletion of IFT20 impairs osteoblast polarity and cell alignment via ceramide-PKC*ζ*-*β*-catenin signaling [96]
IFT140	Osx-Cre;IFT140^fl/fl^ mice exhibited dwarf phenotypes, such as short bone length, less bone mass, and decreased bone mineral apposition rate [21]
IFT80	Osx-Cre;IFT80^fl/fl^ mice show reduced bone mass with impaired osteoblast differentiation; IFT80 is required for osteoblast differentiation by balancing between canonical and noncanonical Hedgehog pathways [11]
KIF3a	Osx-Cre;Kif3a^fl/fl^ mice display an osteopenia phenotype with impaired osteoblast function. Kif3a deletion in osteoblast impairs osteoblast-mediated bone formation through multiple pathways including intracellular calcium, hedgehog, and Wnt signaling [16] Col1-Cre;Kif3a^fl/fl^ mice have normal bone development but reduced bone formation in response to a cyclic ulnar loading [97]
PKD	Osx-Cre;Pkd1^flox/m1Bei^ mice show reduced bone mass, mineral apposition rates, increased adipogenesis in bone marrow, and impaired osteoblast differentiation [19]

**Table 2:** The role of primary cilia in cartilage development illustrated by the conditional knock out mouse model.

Gene	Function
IFT20	Col2-cre;Ift20^fl/fl^ has normal limb development, but Prx-cre;Ift20^fl/fl^ mouse shows four limb development defects. Deletion of Ift20 increased Fgf18 expression in the perichondrium that sustained Sox9 expression, thus preventing endochondral ossification [98]
IFT80	Deletion of IFT80 in the embryonic stage (injected tamoxifen at embryonic day 14.5 in Col2-creERT;IFT80 mouse) shows shortened cartilage and limbs at birth; deletion of IFT80 in the postnatal stage (injected tamoxifen at postnatal day 4 in Col2-creERT;IFT80 mouse) causes reduced growth plate length; loss of IFT80 blocks chondrocyte differentiation by disruption of ciliogenesis and alteration of Hh and Wnt signaling transduction, which in turn alters epiphyseal and articular cartilage formation [99]
IFT88	Col2-Cre;Ift88^fl/fl^ mice display disorganized columnar structure and early loss of growth plate; Ift88 regulates the expression of Sfrp5 and Wnt signaling pathways in the growth plate via regulation of Ihh signaling [9] Aggrecan-CreERT;Ift88^fl/fl^ mice have a thinner articular cartilage thickness in the middle of tibia at 33 weeks old [43]
KIF3a	Col2*α*1-Cre;Kif3a^fl/fl^ mice show postnatal dwarfism with a disorganized growth plate and altered chondrocyte orientation; deletion of Kif3a inhibits cell proliferation but accelerates hypertrophic differentiation, leading to the premature closure of the growth plate [100]
KIF5b	Col2*α*1-Cre;Kif5b^fl/fl^ mice were smaller in stature owing to shortened spine vertebrae and long bones; mutant mice characterized by disorganized columnar structure in the growth plates; Kif5b mutation can cause incomplete cell rotation, proliferation, and differentiation disruption and results in a disorganized growth plate [101]

**Table 3:** Primary cilium-related gene and scoliosis.

Gene	Scoliosis phenotype	Function in cilium biology
TBX6	Congenital and idiopathic scoliosis in humans [68, 69]	Affects morphology and motility of nodal cilia in mice and zebrafish [102, 103]
LBX1	Idiopathic scoliosis association in several ethnic groups, confirmed using different approaches [104–107]	Deleted in a mouse model of the primary ciliary dyskinesia gene [108]
GPR126	Scoliosis in humans and mice [109–111]	Essential for the development of myelinated axons [70, 112]
PAX1	Congenital and idiopathic scoliosis in humans and mice [71–73]	Other family members are associated with cilium signaling pathways [113–115]
POC5	Idiopathic scoliosis in humans [74]	Essential for centriole structure [116, 117]
KIF6	Idiopathic-type curvature in zebrafish [75]	Predicted to be involved in ciliary function or structure [118]
PTK7	Idiopathic-type curvature in zebrafish [76]	Role in cilium orientation in zebrafish [77]
FGF3	Idiopathic scoliosis in a KO mouse model; scoliosis in a human case report carrying loss-of-function mutation in the gene [78, 79]	Affecting the organization of chondrocyte primary cilia in the growth plate in mice [80]
SHP2	Idiopathic scoliosis in a KO mouse model [81, 82]	The length of primary cilia reduced in mutated mice [81]
IFT88	Idiopathic-type curvature in human and zebrafish [64, 83]	Essential for ciliogenesis [83]
IFT20	Idiopathic-type curvature in zebrafish [83]	Essential for ciliogenesis [83]
Arl13b	Idiopathic-type curvature in zebrafish [83]	Essential for ciliogenesis [83]
Yap	Idiopathic-type curvature in zebrafish [83]	Interacts with cilia [83]

## Data Availability

All the data are included within the article.
